# Wake After Sleep Onset Mediates the Link Between Neighborhood Social Environment and Cognition in Older Adults

**DOI:** 10.1093/geroni/igab046.1467

**Published:** 2021-12-17

**Authors:** Bernadette Fausto, Kexin Yu, Paul Duberstein

**Affiliations:** 1 Rutgers University–Newark, Newark, New Jersey, United States; 2 USC, LA, California, United States; 3 Rutgers University School of Public Health, Piscataway, New Jersey, United States

## Abstract

Cognition is influenced by the neighborhood social and physical environment, but the underlying mechanisms by which neighborhood environment affects cognition are unclear. We tested the hypothesis that sleep mediates the effects between environmental exposures and cognition. We employed structural equation modeling to examine interrelationships among neighborhood social and physical environment, actigraphic sleep characteristics, and global cognition in a sample of older adults (N=3,196) from Round 2 of the National Social Life, Health, and Aging Project. Results indicated that participants with better cognition lived in salutary (e.g., cohesive, safe) social environments (est.=0.03, p<.001) and less disruptive (e.g., noisy, polluted) physical environments (est.=-0.04, p<.001). The mediation hypothesis was partially supported. Time spent awake after sleep onset mediated the social environment-cognition relationship, but sleep characteristics did not mediate the physical environment-cognition relationship. Future work should identify other environmental influences on sleep and cognition in aging to inform public health intervention priorities.

